# Efficiency Bounds for Minimally Nonlinear Irreversible Heat Engines with Broken Time-Reversal Symmetry

**DOI:** 10.3390/e21070717

**Published:** 2019-07-23

**Authors:** Qin Liu, Wei Li, Min Zhang, Jizhou He, Jianhui Wang

**Affiliations:** 1 Department of Physics, Nanchang University, Nanchang 330031, China; 2 State Key Laboratory of Surface Physics and Department of Physics, Fudan University, Shanghai 200433, China; 3 State Key Laboratory of Theoretical Physics, Institute of Theoretical Physics, Chinese Academy of Sciences, Beijing 100190, China

**Keywords:** heat engine, nonlinear irreversible, broken time-reversal symmetry, efficiency at maximum power, 05.70.Ln

## Abstract

We study the minimally nonlinear irreversible heat engines in which the time-reversal symmetry for the systems may be broken. The expressions for the power and the efficiency are derived, in which the effects of the nonlinear terms due to dissipations are included. We show that, as within the linear responses, the minimally nonlinear irreversible heat engines can enable attainment of Carnot efficiency at positive power. We also find that the Curzon-Ahlborn limit imposed on the efficiency at maximum power can be overcome if the time-reversal symmetry is broken.

## 1. Introduction

Heat engines as energy converters provide a good platform for studying the nature of thermodynamics, in addition to its relation with utilization of energy resources. Exploring the efficient heat engines at large power is therefore an issue of significance in thermodynamics. The second law of thermodynamics tells us that the efficiency of a heat engine working between two heat reservoirs of constant temperatures $T_{h}$ and $T_{c}$ (<$T_{h})$ is bounded by the Carnot efficiency $\eta_{C}=1-T_{c}/T_{h}$. As the Carnot engine needs infinite time for completing a cycle and produces null power, practically, a heat engine needs to be sped up. Starting with Curzon and Ahlborn model [1], the issue of the efficiency at maximum power and its possible universal bounds was intensively studied in the literature [2,3,4,5,6,7,8,9,10,11,12,13,14,15,16,17,18,19,20,21,22,23,24,25,26,27]. Another increasingly interesting topic is the attainable maximum efficiency at nonvanishing power for the heat engines and it has attracted much attention recently [28,29,30,31,32,33,34,35,36,37].

In the seminal paper [28] the bounds on efficiency for a specific model of steady state heat engine with broken time-reversal symmetry caused, for example, by an external magnetic field were investigated. It was shown that, within the linear response regime, this time-reversal antisymmetry can significantly boost the performance and, in principle, enable attainment of Carnot limit at nonzero power. The performance of the steady state heat engine working in the linear response regime, with broken time-reversal symmetry, raised issues that deserve to be addressed. For instance, is there improvement of performance in cyclic heat engines induced by broken time-reversal symmetry? Can heat engines beyond the linear response regime allow the Carnot limit at positive power, with or without broken time-reversal symmetry? How to identify the relations between the power, efficiency, and unavoidable dissipations? The broken time-reversal symmetry was found to boost the performance of cyclic heat engines in the linear responses [21,32]. The general relations between the efficiency, power and dissipations were analyzed in the regimes of linear [19,38,39,40,41,42] and nonlinear [43] responses. Although approaching Carnot efficiency at finite power is not accessible under some specific conditions [38,41,44,45], it can be achieved in certain limits for the cyclic or steady state heat engines [19,33,34,36,37,46]. It was found that systems [33,34,46] with phase transitions or a singular transport law enable the realization of Carnot limit at nonzero power, even beyond linear response regime. Recent theoretical studies on efficiency at maximum power and any deviation from efficiency at maximum power of the nonlinear irreversible heat engine, with and without broken time-reversal symmetry, have been present under the assumption of minimally nonlinear irreversible thermodynamics (in which the restriction on Onsager coefficients due to the nonnegative entropy production rate derived from the linear response, however, was still approximately borrowed from nonlinear case) [18,26]. Nevertheless, a unified description of the performance at maximum efficiency and maximum power for nonlinear irreversible heat engines, which are based on exact conditions of Onsager coefficients imposed by the second law of thermodynamics and where the time-reversal symmetry could be broken, is still lacking. Since an analytical analysis on Onsager coefficients in the nonlinear thermodynamics [47] is complicated, the study of nonlinear irreversible heat devices without any approximation must resort to numerical calculations (see, for example, [48,49,50]). Fortunately, a minimally nonlinear assumption, first posed in Ref. [26], has been widely used and tested in various nonlinear irreversible heat devices [17,18,43,51,52]. For this reason, we proposed a unified analytical approach for minimally nonlinear irreversible heat engines with broken time-reversal symmetry, with special analysis on the attainment of Carnot efficiency at nonvanishing power.

In the present paper, we investigate the questions of whether the maximum efficiency can approach the Carnot limit at positive power and whether the Curzon–Ahlborn limit for the efficiency at maximum power can be exceeded in the nonlinear response regime. We propose a minimally nonlinear irreversible heat engine [26], in which the nonlinear regime is included [26,51], and study its efficiency and power for the case of broken time-reversal symmetry. We show that the maximum efficiency can reach the Carnot value at nonzero power and the Curzon–Ahlborn limit on the efficiency at maximum power is overcome in the time-reversal antisymmetry.

## 2. Minimally Nonlinear Irreversible Heat Engine with Broken Time-Reversal Symmetry

The heat engine model under consideration, which may be cyclic or steady state and where broken time-reversal symmetry may be induced, for instance, by interaction with an external magnetic field B. The working substance is in contact with a hot reservoir and a cold one of temperatures $T_{h}$ and $T_{c}$ (<$T_{h}$). In order to describe the minimally nonlinear irreversible heat engines in which only a second-order nonlinear term is added in the linear Onsager relations to describe the nonlinear case, we adopt the extended Onsager relations [26,51] with inclusion of external field B,

(1) $$J_{1}\left(B\right)=L_{11}\left(B\right)X_{1}+L_{12}\left(B\right)X_{2},$$

(2) $$J_{2}\left(B\right)=L_{21}\left(B\right)X_{1}+L_{22}\left(B\right)X_{2}-\gamma_{h}J_{1}^{2}\left(B\right),$$

where the nonlinear term $\gamma_{h}J_{1}^{2}$ denotes heat dissipation into the hot reservoir and $\gamma_{h}\;(\geq$0) indicates the dissipation strength. The linear response is recovered by setting $\gamma_{h}=0$ in Equation (2) and it indicates $X_{1}\to 0$ and $X_{2}\to 0$. However, the nonlinear response described by Equations (1) and (2) is not restricted to small values of $X_{1}$ and $X_{2}$. Noteworthy, the time-reversal symmetry will be broken due to the external field B, thereby leading to the Onsager coefficients $L_{12}\left(B\right)\neq L_{21}\left(B\right)$ for the heat engines under consideration, though the Onsager–Casimir relation $L_{12}\left(B\right)=L_{21}\left(-B\right)$ is satisfied. For sake of convenience, the following formula will include the external field but without explicitly writing B.

In the heat engine, the heat flux ${\dot{Q}}_{h}$ is extracted from the hot heat reservoir at the temperature $T_{h}$, and there must be a certain heat current ${\dot{Q}}_{c}$ injected to the cold heat reservoir of temperature $T_{c}$, with corresponding production of power output $P={\dot{Q}}_{h}-{\dot{Q}}_{c}$. Throughout the paper the dot means the quantity per unit time for steady-state heat engines or the quantity divided by the cycle time duration for cyclic machines. Since the entropy production of a steady-state or a cyclic heat engine merely contributed from the two heat reservoirs, and its rate thus reads

(3) $$\dot{\sigma }=-\left(\frac{\dot{Q_{h}}}{T_{h}}-\frac{\dot{Q_{c}}}{T_{c}}\right)=-\frac{P}{T_{c}}+\dot{Q_{h}}\left(\frac{1}{T_{c}}-\frac{1}{T_{h}}\right).$$

Without loss of generality, the power output *P* can be expressed as $P=F\dot{x}$, where *F* is an external force and *x* is its corresponding thermodynamically conjugate variable. As the entropy production rate can be expressed in terms of the thermodynamic fluxes J and forces X: $\dot{\sigma }=JX$, from Equation (3) we have

(4) $$\dot{\sigma }=J_{1}X_{1}+J_{2}X_{2}$$

through defining the thermodynamic fluxes $J_{1}\equiv \dot{x}$ and $J_{2}\equiv \dot{Q_{h}}$, with conjugate affinities $X_{1}=F/T_{c}$ and $X_{2}=1/T_{c}-1/T_{h}$. The power output can thus be expressed as

(5) $$P=-J_{1}X_{1}T_{c}.$$

Based on Equations (1) and (2), we can rewrite $J_{2}$ as

(6) $$J_{2}=\frac{L_{21}}{L_{11}}J_{1}+L_{22}\left(1-\frac{L_{12}L_{21}}{L_{11}L_{22}}\right)X_{2}-\gamma_{h}J_{1}^{2}.$$

Let $J_{3}\equiv {\dot{Q}}_{c}$, we have $J_{3}=\dot{Q_{h}}-P=J_{1}X_{1}T_{c}+J_{2},$ which takes the form of

(7) $$J_{3}=\frac{L_{21}-L_{12}X_{2}T_{c}}{L_{11}}J_{1}+L_{22}\left(1-\frac{L_{12}L_{21}}{L_{11}L_{22}}\right)X_{2}-\gamma_{c}J_{1}^{2},$$

where $\gamma_{c}=T_{h}/L_{11}-\gamma_{h}$ has been used. Here $\gamma_{c}$ represents the strength of the heat dissipation along the cold heat-transfer process and $\gamma_{c}$ becomes $\gamma_{c}=T_{h}/L_{11}$ in the linear response. We emphasize that the nonlinear term $\gamma_{h}J_{1}^{2}$ in Equation (6) becomes vanishing either in the quasistatic limit or in the linear response. Unlike in the linear irreversible thermodynamics where the temperature gradient $T_{h}-T_{c}$ must be smaller than the temperatures $T_{h}$ and $T_{c}$ of the reservoirs, the nonlinear irreversible thermodynamics as a direct expansion by including nonlinear terms $\gamma_{h,c}J_{1}^{2}$ takes into account the irreversibility induced by finite operation time and finite temperature difference. That is, the nonlinear term $\gamma J_{1}^{2}$ is inevitable, existing either in the finite-time operation or in the finite temperature difference, and it therefore indicates a higher degree of nonequilibrium compared to the linear response.

With consideration of Equations (1), (4) and (6), we find that the Onsager coefficients must be constrained by

(8) $$L_{11}\geq 0,\;L_{22}\geq 0,\;L_{11}L_{22}-L_{11}L_{22}\alpha \eta_{C}-{\left(L_{12}+L_{21}\right)}^{2}/4+L_{12}L_{21}\alpha \eta_{C}\geq 0,$$

due to the nonnegativity of the entropy production rate ($\dot{\sigma }\geq 0)$. Here and hereafter we define $\alpha \equiv 1/(1+\gamma_{c}/\gamma_{h})$ and take α rather than $\gamma_{c}/\gamma_{h}$ as the dissipation ratio for simplicity. The asymmetric dissipation limits $\gamma_{c}/\gamma_{h}\to \infty$ and $\gamma_{c}/\gamma_{h}\to 0$ correspond to α=0 and α=1, respectively. The symmetrical dissipation case when $\gamma_{h}=\gamma_{c}$ leads to α=1/2. When the entropy production rate tends to be zero ($\dot{\sigma }=0$), we have $L_{11}L_{22}-L_{11}L_{22}\alpha \eta_{C}-{\left(L_{12}+L_{21}\right)}^{2}/4+L_{12}L_{21}\alpha \eta_{C}=0.$

## 3. Maximum Efficiency

As the efficiency η takes the form of

(9) $$\eta =\frac{P}{J_{2}}=\frac{-J_{1}X_{1}T_{c}}{L_{21}X_{1}+L_{22}X_{2}-\gamma_{h}{\left(L_{11}X_{1}+L_{12}X_{2}\right)}^{2}}.$$

The derivation of η with respect to $X_{1}$ gives rise to the expression of the maximum efficiency,

(10) $$\eta_{\max}=\eta_{C}\frac{y+2-2\sqrt{y+1-\alpha \eta_{C}yx}}{4\alpha \eta_{C}+y/x},$$

at the thermodynamic force

(11) $$X_{1}=\frac{L_{11}X_{2}\left(L_{22}-L_{12}^{2}X_{2}\gamma_{h}\right)-\sqrt{L_{11}\left(L_{11}L_{22}-L_{12}L_{21}\right)X_{2}^{2}\left(L_{22}-L_{12}^{2}X_{2}\gamma_{h}\right)}}{L_{11}\left(-L_{21}+L_{11}L_{12}X_{2}\gamma_{h}\right)},$$

where we have introduced two parameters $x=L_{12}/L_{21},$ and $y=L_{12}L_{21}/\left(L_{11}L_{22}-L_{12}L_{21}\right).$

Since no restriction is imposed on the attainable values of the asymmetry parameter *x*, the relation (8) yields

$$\left\{ \begin{array}{c} g(x)\leq y\leq 0\;\;\;\;\;(x\leq 0),\;\;\;\;\;\;\;\;\;\;\;\;\;\;\;\;\;\;\;\;\;\;(12a)\\ 0\leq y\leq g(x)\;\;\;\;\;(x>0),\;\;\;\;\;\;\;\;\;\;\;\;\;\;\;\;\;\;\;\;\;\;(12b) \end{array}\right.$$

where we have defined

(13) $$g\left(x\right)\equiv \frac{4(1-\alpha \eta_{C})x}{{\left(x-1\right)}^{2}}.$$

**Figure 1:**
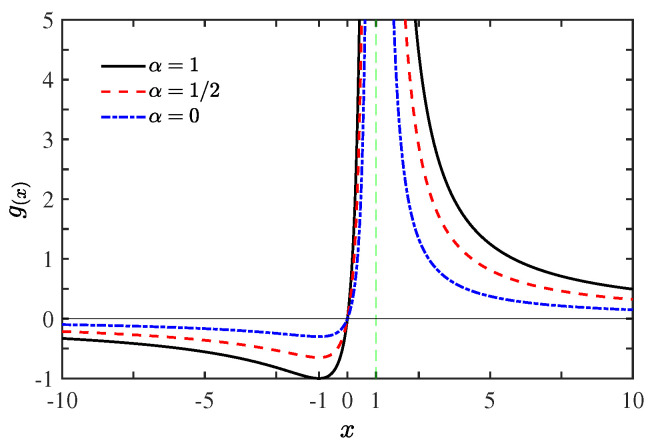
The function g(x) as a function of the asymmetry parameter *x*, with dissipation parameter α=1 (black solid line), α=1/2 (red dashed line), and α=0 (blue dot-dashed line). The vertical asymptote of g(x) at x=1 is indicated by green dotted line (when α≠0, $\eta_{C}=0.7$ is adopted).

It reduces to $g\left(x\right)=4x/{\left(x-1\right)}^{2}$ obtained in the linear response regime [28], if the dissipation vanishes $\gamma_{h}\to 0$ as well as α→0. We stress that direct use of $g\left(x\right)=4x/{\left(x-1\right)}^{2}$ as done in Ref. [18] would yield nonphysical, negative entropy production rate for the nonlinear case with α≠0. The effects of nonvanishing dissipation (α≠0) on the bound function g(x) are of significance for any *x*, as shown in Figure 1. For a given asymmetry parameter *x*, the maximum value $\eta_{M}$ of Equation (10) is achieved if y=g(x). Considering Equations (12) and (13), we can obtain the maximum efficiency $\eta_{M}$ via simple algebra as follows: when $\alpha \eta_{C}\leq 1/2$,

$$\eta_{M}=\{\begin{array}{ccc} \eta_{C}\frac{x^{2}\left(1-\alpha \eta_{C}\right)}{\left(x-2\right)x\alpha \eta_{C}+1} & \left(\frac{1}{2\alpha \eta_{C}-1}\leq x\leq 1\right), & \;\;\;\;\;\;\;\;\;\;\;\;\;\;\;\;\;\;\;\;\;\;(14a)\\ \eta_{C} & \left(x\leq \frac{1}{2\alpha \eta_{C}-1}\;\mathrm{and}\;x\geq 1\right), & \;\;\;\;\;\;\;\;\;\;\;\;\;\;\;\;\;\;\;\;\;\;(14b) \end{array}$$

and when $1/2<\alpha \eta_{C}\leq 1$,

$$\eta_{M}=\{\begin{array}{ccc} \eta_{C}\frac{x^{2}\left(1-\alpha \eta_{C}\right)}{\left(x-2\right)x\alpha \eta_{C}+1} & \left(x\geq \frac{1}{2\alpha \eta_{C}-1}\;\mathrm{and}\;x\leq 1\right), & \;\;\;\;\;\;\;\;\;\;\;\;\;\;\;\;\;\;\;\;\;\;\;\;\;\;\;\;\;(15a)\\ \eta_{C} & \left(1\leq x\leq \frac{1}{2\alpha \eta_{C}-1}\right). & \;\;\;\;\;\;\;\;\;\;\;\;\;\;\;(15b) \end{array}$$

**Figure 2:**
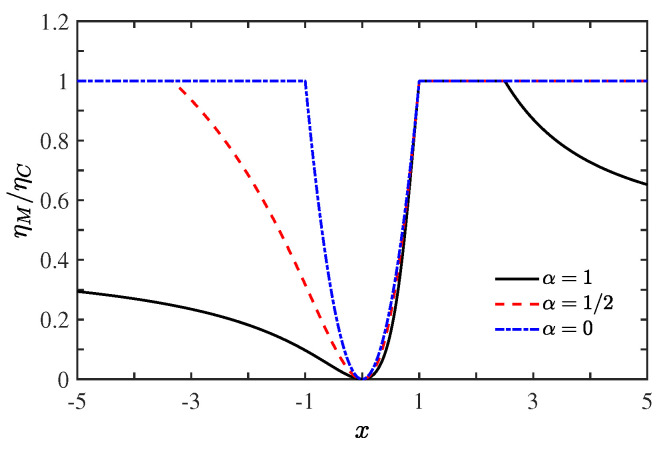
(Color online) Ratio $\eta_{M}/\eta_{C}$ as a function of the asymmetry parameter *x*. The dissipation ratios are α=1 (black solid line), α=1/2 (red dashed line), and α=0 (blue dot-dahsed line) (when α≠0, $\eta_{C}=0.7$ is adopted).

If, in particular, α→0 as the dissipation vanishes $\gamma_{h}\to 0$, Equations (14b) and (14a) simplify to

$$\eta_{M}=\{\begin{array}{ccc} \eta_{C}x^{2} & \left(|x|\leq 1\right), & \;\;\;\;\;\;\;\;\;\;\;\;\;\;\;\;\;\;\;\;\;\;(16a)\\ \eta_{C} & \left(|x|\geq 1\right), & \;\;\;\;\;\;\;\;\;\;\;\;\;\;\;\;\;\;\;\;\;\;(16b) \end{array}$$

which were obtained within the framework of linear irreversible thermodynamics [28,38]. Besides $\eta_{C}$, the function depends on $\eta_{M}$ both *x* and α if the dissipation exists with α≠0. For $\alpha \leq {\left(2\eta_{C}\right)}^{-1}$, the Carnot efficiency can be approached when x≥1 and when $x\leq {\left(2\alpha \eta_{C}-1\right)}^{-1}$; whereas for ${\left(2\eta_{C}\right)}^{-1}<\alpha \leq \eta_{C}^{-1}$, the range in which the Carnot limit is reached becomes $1\leq x\leq {\left(2\alpha \eta_{C}-1\right)}^{-1}$. The ratio $\eta_{M}/\eta_{C}$ for different values of α is drawn in Figure 2, where $\eta_{C}=0.7$ for α≠0 is adopted. Let us consider two special cases: (1) when α=1 and thus $\alpha \eta_{C}=0.7$, the Carnot limit is reached during the range of 1≤x≤2.5, and $\eta_{M}=\eta_{C}\left\{3x^{2}/\left[1+0.7\left(x-2\right)x\right]\right\}$ when x≥2.5 or x≤1; (2) when α=1/2 and $\alpha \eta_{C}=0.35$, the Carnot limit is obtained in the region of x≤−3.33 and x≥1. The former and latter cases are indicted by the black solid line and the red dashed one, respectively, in Figure 2 where the linear irreversible case (α=0) is represented by the blue dot-dashed line. Since the Carnot efficiency is obtained under the condition y=g(x), we find that $\det\left(L\right)={\left(L_{12}-L_{21}\right)}^{2}/\left[4\left(1-\alpha \eta_{C}\right)\right]$, and the entropy production rate $\dot{\sigma }=0$. The Carnot limit and $L_{12}\neq L_{21}$ yields det(L)>0, showing that the Carnot efficiency could be realized only in the non-tight coupling case.

We find from Equations (5) and (11) that the power at maximum efficiency reads

(17) $$P_{m\eta }=\eta_{C}X_{2}L_{21}^{2}\frac{|\left(x-1\right)\left[\left(1-2\alpha \eta_{C}\right)x+1\right]|{\left(|x-1|-|\left(1-2\alpha \eta_{C}\right)x+1|\right)}^{2}}{16L_{11}{\left(1-\alpha \eta_{C}\right)}^{2}{\left(1-\alpha \eta_{C}x\right)}^{2}},$$

which is always positive and simplifies for $0\leq \alpha \eta_{C}\leq 1/2$ and $1/2<\alpha \eta_{C}\leq 1$ to

$$P_{m\eta }=\{\begin{array}{ccc} \eta_{C}x^{2}X_{2}L_{21}^{2}\frac{\left(1-x\right)[\left(1-2\alpha \eta_{C}\right)x+1]}{4L_{11}{\left(1-\alpha \eta_{C}x\right)}^{2}} & \left(\frac{1}{2\alpha \eta_{C}-1}\leq x\leq 1\right), & \;\;\;\;\;\;\;\;\;\;\;\;\;\;\;\;\;\;\;\;\;\;(18a)\\ \eta_{C}X_{2}L_{21}^{2}\frac{\left(x-1\right)[\left(1-2\alpha \eta_{C}\right)x+1]}{4L_{11}{\left(1-\alpha \eta_{C}\right)}^{2}} & \left(x\leq \frac{1}{2\alpha \eta_{C}-1}\;\mathrm{and}\;x\geq 1\right), & \;\;\;\;\;\;\;\;\;\;\;\;\;\;\;\;\;\;\;\;\;\;(18b) \end{array}$$

and

$$P_{m\eta }=\{\begin{array}{ccc} \eta_{C}x^{2}X_{2}L_{21}^{2}\frac{\left(1-x\right)[\left(1-2\alpha \eta_{C}\right)x+1]}{4L_{11}{\left(1-\alpha \eta_{C}x\right)}^{2}} & \left(x\geq \frac{1}{2\alpha \eta_{C}-1}\;\mathrm{and}\;x\leq 1\right), & \;\;\;\;\;\;\;\;\;\;\;\;\;\;\;\;\;\;\;\;\;\;(19a)\\ \eta_{C}X_{2}L_{21}^{2}\frac{\left(x-1\right)[\left(1-2\alpha \eta_{C}\right)x+1]}{4L_{11}{\left(1-\alpha \eta_{C}\right)}^{2}} & \left(1\leq x\leq \frac{1}{2\alpha \eta_{C}-1}\right), & \;\;\;\;\;\;\;\;\;\;\;\;\;\;\;\;\;\;\;\;\;\;(19b) \end{array}$$

respectively. From Equations (14b), (15b), (18b), and (19b), we see that for $0\leq \alpha \eta_{C}\leq 1/2$ the Carnot efficiency is attained at positive power in the range of x≥1 and $x\leq {\left(1-2\alpha \eta_{C}\right)}^{-1}$, and that for $1/2\leq \alpha \eta_{C}\leq 1$ the Carnot limit can also be reached with nonzero power if $1\leq x\leq {\left(2\alpha \eta_{C}-1\right)}^{-1}$. The special case of the linear response regime when α=0 results into the fact that the Carnot efficiency is achieved only when |x|≥1, as expected. We emphasize here that the nonzero power at the Carnot efficiency is found by using y=g(x), which implies vanishing entropy production rate ($\dot{\sigma }=0$).

## 4. Efficiency at Maximum Power

We now turn to the maximum power output $P_{\max}$ and its corresponding efficiency $\eta_{mp}$. It follows, using Equation (5) and setting $\partial P/\partial X_{1}=0$, that the power output achieves its maximum value,

(20) $$P_{\max}=\frac{\eta_{C}L_{12}^{2}}{4L_{11}}X_{2}$$

at

(21) $$X_{1}=-\frac{L_{12}}{2L_{11}}X_{2}.$$

Substituting Equation (21) into Equation (9), we find that the efficiency at maximum power is

(22) $$\eta_{mp}=\frac{\eta_{C}}{2}\frac{2xy}{4+y(2-x\alpha \eta_{C})},$$

whose upper bound $\eta_{mp}^{*}$ is obtained when and only when y=g(x). By substitution of Equation (12) into Equation (22) we then arrive at

(23) $$\eta_{mp}^{*}=\eta_{C}\frac{1-\alpha \eta_{C}}{{\left(\alpha \eta_{C}-x^{-1}\right)}^{2}-\alpha \eta_{C}+1}.$$

**Figure 3:**
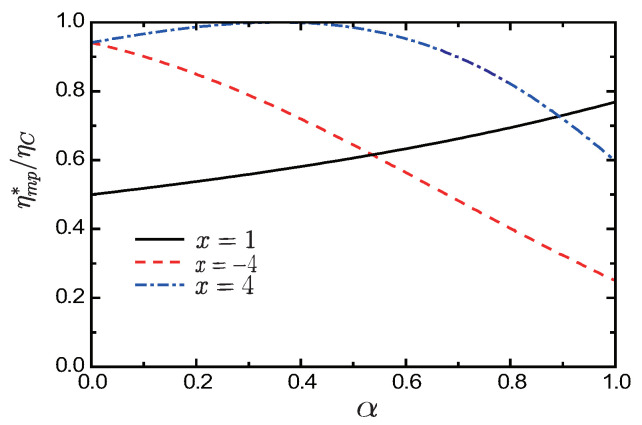
(Color online) Ratio $\eta_{mp}^{*}/\eta_{C}$ as a function of the dissipation ratio α, with asymmetric parameters x=1 (black solid line), x=−4 (red dashed line) and x=4 (blue dot-dashed line) ($\eta_{C}=0.7$ is adopted).

**Figure 4:**
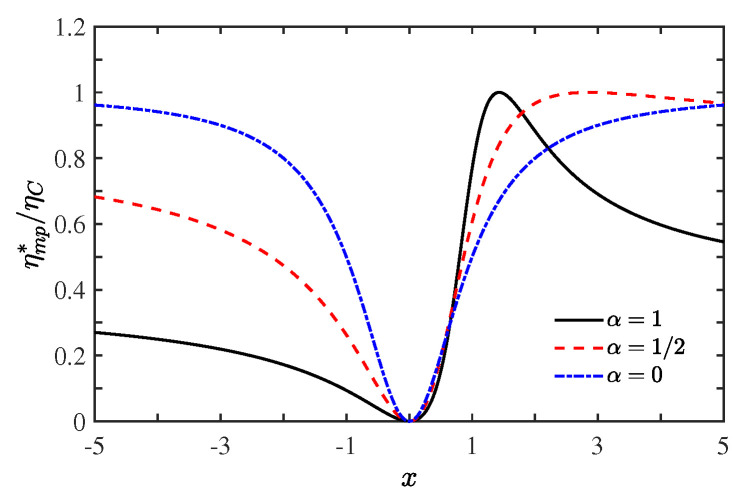
(Color online) Ratio $\eta_{mp}^{*}/\eta_{C}$ as a function of the asymmetry parameter *x*, with dissipation ratios α=1 (black solid line), α=1/2 (red dashed line) and α=0 (blue dot-dashed line) (when α≠0, $\eta_{C}=0.7$ is adopted).

To see how the time-broken asymmetry induced by the external field influences the performance on the heat engine, in Figure 3 we plot dimensionless maximum power output ($\eta_{mp}^{*}/\eta_{C}$) versus dissipation ratio (α) for different values of asymmetry parameter α, with x=1 (black solid line), x=−4 (red dashed line), and x=4 (blue dot-dashed line). Figure 3 shows that the efficiency at maximum power $\eta_{mp}^{*}$ (as a function of α) depends sensitively on the asymmetry parameter *x*. When x=−4, the optimal efficiency $\eta_{mp}^{*}$ monotonically decreases with increasing dissipation ratio α. For x=4, the curve of $\eta_{mp}^{*}$ versus α is of parabolic shape and its maximum value $\eta_{mp}^{*}=\eta_{C}$ is located at $\alpha =1/(4\eta_{C})$. In the absence of the external magnetic field or in the symmetric case (x=1), the optimal efficiency $\eta_{mp}^{*}$ is a monotonically increasing function of the dissipation ratio α. We note that, for $x=1/(\alpha \eta_{C})$ or |x|→∞, $\eta_{mp}^{*}=\eta_{C}$, so the Carnot efficiency $\eta_{C}$ and the maximum power $P_{max}$ can be attained simultaneously. It is therefore indicated that the limit imposed on the efficiency at maximum power for systems with time-reversal symmetry is overcome in the systems without this symmetry. If nonlinear term vanishes ($\gamma_{h}\to 0$ and α→0), the efficiency at maximum power $\eta_{mp}^{*}=\eta_{C}x^{2}/\left(1+x^{2}\right)$ in the linear situation is recovered and $\eta_{mp}^{*}\to \eta_{C}$ as |x|→∞. Figure 4 shows that the efficiency at maximum power $\eta_{mp}^{*}$ (for given α) expressed by Equation (23). Insight can be gained into the condition of attainment of the Carnot efficiency by seeing first from Figure 4 that for α=1 and $\eta_{C}=0.7$ efficiency at maximum power $\eta_{mp}^{*}=\eta_{C}$ can be achieved at the point x=1/0.7≃1.428 (or |x|→∞ which is not shown in the figure). Second, from Figure 4, we note that for x<0 the efficiency $\eta_{mp}^{*}$ increases more slowly to approach the Carnot limit in the minimally nonlinear response regime than in the linear response case.

We emphasize that, for the time-reversal symmetry (x=1), the efficiency at maximum power (23) becomes

(24) $$\eta_{mp}^{*}=\frac{\eta_{C}}{2-\alpha \eta_{C}},$$

which is situated between $\eta_{C}/2\leq \eta_{mp}^{*}\leq \eta_{C}/\left(2-\eta_{C}\right)$ as 0≤α≤1. The upper bounds and lower bounds were obtained earlier in the low-dissipation Carnot heat engines [23] and the minimally nonlinear irreversible heat engines [26,52] satisfying the tight-coupling condition at the asymmetrical dissipation limits. In accordance with a linear response theory where α=0, the linear coefficient of the expansion of is expected to be $\eta_{mp}^{*}=\eta_{C}/2$ [20]. In the dissipation symmetric limit α=1/2, we find that the maximum efficiency at maximum power is $\eta_{mp}^{*}=\eta_{C}/\left(2-\eta_{C}/2\right)$, and its expansion in terms of $\eta_{C}$ up to third order is $\eta_{mp}^{*}=\eta_{C}/2+\eta_{C}^{2}/8+3\eta_{C}^{3}/32+O\left(\eta_{C}^{4}\right)$, which agrees well with the expansion of the famous Curzon–Ahlborn efficiency, $\eta_{CA}=1-\sqrt{T_{c}/T_{h}}=\eta_{C}/2+\eta_{C}^{2}/8+\eta_{C}^{3}/16+O\left(\eta_{C}^{4}\right)$, indicating that they have the same universality of $\eta_{C}/2+\eta_{C}^{2}/8$.

**Figure 5:**
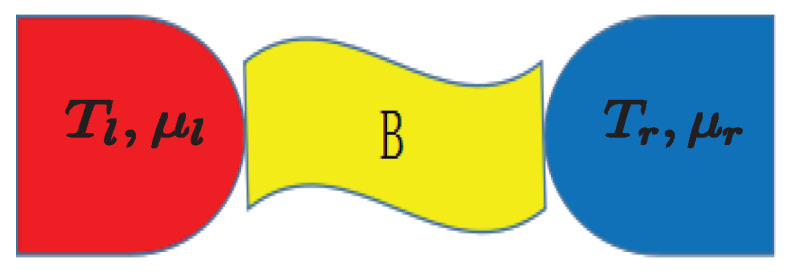
(Color online) The schematic diagram of the two-terminal thermoelectric model.

Figure 4 shows that, for x>0, the efficiency at maximum power achieves Carnot efficiency faster, due to lager fluctuations [37] in the nonlinear response regime than in the linear responses. A two-terminal thermoelectric device with broken time-reversal symmetry can be exemplified in our model, as sketched in Figure 5. An external magnetic filed (such as a probe [53]) in contact with both the right and left reservoirs is introduced in order for the time-reversal symmetry to be broken with $L_{12}\neq L_{21}$, but under the constraints that the average thermal and electrical currents extracted from this external setup (ex) are zero via controlling the the temperature $T_{ex}$ and chemical potential $\mu_{ex}$. The thermodynamic fluxes for such a thermoelectric device the electrochemical potential $X_{1}=\left(\mu_{l}-\mu_{r}\right)/\left(eT_{r}\right)$ and the temperature difference $X_{2}=1/T_{r}-1/T_{l}$, where *e* is the electronic charge, $T_{l,r}$ and $\mu_{l,r}$ denote the temperatures and chemical potentials in the left (*l*) and right (*r*) electronic reservoirs. Specifically, at the time-reversal symmetry with x=1, the nonlinear terms ($\gamma J_{1}^{2}$) indicating higher degree of nonequilibrium [26] enhance the performance of the heat engine via improving the efficiency at maximum power compared to the linear response, which agrees well with that found in thermoelectric engines [48,49,50]. We also stress that, since not the degree of nonequilibrium but also the symmetry parameter *x* can affect the efficiency and power, for some values of *x* with x≠1, $\eta_{mp}\left(x\neq 1\right)$ can be smaller in the nonlinear response than in the linear responses(see Figure 4).

## 5. Conclusions

For systems with broken time-reversal symmetry, we have investigated the performance of minimally nonlinear irreversible heat engines (based on these systems). For these nonlinear irreversible heat engines, the maximum efficiency can tend to be the Carnot limit at nonzero power and efficiency at maximum power can go beyond the Curzon–Ahlborn limit when the asymmetric parameter *x* satisfies a certain condition. We pointed out that a two-terminal thermoelectric device with broken time-reversal symmetry can be mapped onto the engine model discussed here. Our analytical results provide a theoretical framework for understanding of minimally nonlinear heat engines, but should also be helpful for studying the heat devices in which higher nonlinear terms due to dissipations are involved.

## Abbreviations

Symbol	Description
$T_{h}$	Temperature of the hot heat reservoir
$T_{c}$	Temperature of the cold heat reservoir
${\dot{Q}}_{h}$	Heat current extracted from the hot reservoir
${\dot{Q}}_{c}$	Heat current injected into the cold reservoir
$\dot{\sigma }$	Entropy production rate
$J_{i}$ with i=1,2,3	Thermodynamic fluxes
$X_{i}$ with i=1,2	Thermodynamic forces
η	Efficiency
$\eta_{C}$	Carnot efficiency
$\eta_{M}$	Maximum efficiency
$\eta_{mp}^{*}$	The upper bound of efficiency at maximum power
*P*	Power output
$P_{\max}$	Maximum power
$P_{m\eta }$	Power at maximum efficiency
α	Dissipation ratio
B	External field
*x*	Asymmetry parameter
